# The application prospects of robot pose estimation technology: exploring new directions based on YOLOv8-ApexNet

**DOI:** 10.3389/fnbot.2024.1374385

**Published:** 2024-04-05

**Authors:** XianFeng Tang, Shuwei Zhao

**Affiliations:** ^1^Physical Education Department, Zhejiang Wanli University, Ningbo, China; ^2^Physical Education Department, Hebei University of Technology, Tianjin, China

**Keywords:** service robots, human motion pose estimation, YOLOv8-ApexNet, bidirectional routing attention, generalized feature

## Abstract

**Introduction:**

Service robot technology is increasingly gaining prominence in the field of artificial intelligence. However, persistent limitations continue to impede its widespread implementation. In this regard, human motion pose estimation emerges as a crucial challenge necessary for enhancing the perceptual and decision-making capacities of service robots.

**Method:**

This paper introduces a groundbreaking model, YOLOv8-ApexNet, which integrates advanced technologies, including Bidirectional Routing Attention (BRA) and Generalized Feature Pyramid Network (GFPN). BRA facilitates the capture of inter-keypoint correlations within dynamic environments by introducing a bidirectional information propagation mechanism. Furthermore, GFPN adeptly extracts and integrates feature information across different scales, enabling the model to make more precise predictions for targets of various sizes and shapes.

**Results:**

Empirical research findings reveal significant performance enhancements of the YOLOv8-ApexNet model across the COCO and MPII datasets. Compared to existing methodologies, the model demonstrates pronounced advantages in keypoint localization accuracy and robustness.

**Discussion:**

The significance of this research lies in providing an efficient and accurate solution tailored for the realm of service robotics, effectively mitigating the deficiencies inherent in current approaches. By bolstering the accuracy of perception and decision-making, our endeavors unequivocally endorse the widespread integration of service robots within practical applications.

## 1 Introduction

With the continuous progress of technology, service robots, as intelligent systems that integrate various perceptual modes, are becoming increasingly popular in today's society (Sun et al., 2019; Cheng et al., 2020). These robots can not only receive and process visual data but also integrate information from various sensors, such as sound and force, enabling outstanding performance in various complex environments and tasks. The widespread applications of service robots span across fields such as healthcare, manufacturing, and service robots, providing people with more intelligent and flexible solutions (Iskakov et al., 2019; Sattler et al., 2019; Ke et al., 2023). Deep learning technology plays a pivotal role in this field, providing strong support for the performance improvement of service robots. Deep learning algorithms, especially structures like Convolutional Neural Networks (CNN) and Recurrent Neural Networks (RNN), learn feature representations of large amounts of complex data, enabling service robots to more accurately understand and process information from different sensors (Moon et al., 2020; Zhao et al., 2023). This deep learning representation of data helps enhance the robot's perceptual capabilities, thereby strengthening its decision-making and task-execution abilities. Despite the significant improvements brought by deep learning to service robots, there are still challenges and shortcomings in practical applications (Jin et al., 2022). One of them is the accurate estimation of human body movement posture, a crucial aspect in various application scenarios of service robots. In many tasks, such as human-robot collaboration and health monitoring, precise understanding of human body movement posture is essential for effective interaction with humans. Therefore, research on the estimation of human body movement posture has become an urgent and challenging task in the current field of service robots (Boukhayma et al., 2019; Wang et al., 2019; Ji and Zhang, 2023). In this paper, we will focus on exploring methods for motion keypoint detection and quality assessment based on service robots to address the current shortcomings in the estimation of human body movement posture.

In the past few years, several remarkable models have emerged in the field of human body posture assessment, playing a crucial role in enhancing the understanding of human body movements by service robots. The following are five related human body posture assessment models that have garnered widespread attention in recent years:

OpenPose is an open-source human body posture estimation system based on convolutional neural networks, renowned for its end-to-end training framework. By simultaneously detecting multiple key points, including the head, hands, and body, OpenPose is capable of providing robust posture estimation in scenarios with high real-time requirements. However, OpenPose may have certain limitations when dealing with complex occlusions and multi-person scenes (Chen et al., 2020).

HRNet adopts high-resolution input images and effectively preserves both local and global posture information by constructing a multi-scale feature pyramid network. Compared to some low-resolution models, HRNet has achieved a significant improvement in accuracy. However, due to the higher computational cost associated with high-resolution inputs, its real-time performance may be subject to some impact (Li Y. et al., 2020).

AlphaPose is a human body posture estimation model that utilizes a multi-stage cascade network, refining the positions of key points through iterative stages. It emphasizes fine-grained processing for posture estimation, enabling excellent performance in complex scenarios. However, the model may not perform well in situations with rapidly changing postures (Fang et al., 2022).

SimpleBaseline employs a simple yet effective approach by predicting key points through stacking multiple residual blocks. Its lightweight design allows for satisfactory performance even in resource-constrained environments. Nevertheless, SimpleBaseline may have some limitations when dealing with occlusions and complex movements (Zeng et al., 2022).

MuPoTS-3D is a multi-camera-based human 3D pose estimation model with robust cross-camera generalization capabilities. The model, by integrating information from multiple cameras, offers more comprehensive pose information. However, due to the need for collaborative action among multiple cameras, its complexity in practical applications may be relatively high (Shen et al., 2022).

These models signify a progression from traditional to deep learning, from single-scale to multi-scale, and from two-dimensional to three-dimensional approaches (Pillai et al., 2019). While each model has attained considerable success in the domain of human body posture assessment, they also possess their own limitations, raising more intricate questions for real-time motion keypoint detection and quality assessment in service robots. In response to these challenges, we introduce YOLOv8-ApexNet.

YOLOv8-ApexNet not only extends the You Only Look Once (YOLO) series of models but also introduces innovative designs tailored to the requirements of service robots. Specifically, we have integrated two key components: Bidirectional Routing Attention (BRA) and Generalized Feature Pyramid Network (GFPN). Firstly, compared to traditional models, ApexNet significantly enhances real-time performance, enabling faster detection and quality assessment of motion keypoints. Secondly, the model's adaptability in complex scenarios has been strengthened, particularly demonstrating more stable performance in situations involving occlusion and rapid motion changes. Most importantly, ApexNet exhibits higher robustness in real-world applications of service robots, enabling them to understand human body movements more accurately and participate more intelligently in collaborative tasks or service provision.

The contributions of this paper are outlined as follows:

- This paper introduces the YOLOv8-ApexNet model, which is not only an extension of the YOLO series but also incorporates innovative designs into the original framework. By introducing Bidirectional Routing Attention and Generalized Feature Pyramid Network, this model demonstrates higher accuracy and robustness in the tasks of motion keypoint detection and quality assessment for service robots. This provides a more advanced solution for the field of service robots to better understand human body movements accurately.
- The introduction of YOLOv8-ApexNet and the integration of Bidirectional Routing Attention and Generalized Feature Pyramid Network collectively contribute to improving the real-time performance and computational efficiency of service robots systems. Through adopting a lightweight design and efficient information extraction methods, the model reduces computational burden while maintaining high accuracy, achieving more efficient real-time motion keypoint detection and quality assessment. This provides robust support for service robots tasks in practical application scenarios that demand high real-time requirements.
- The introduction of YOLOv8 ApexNet also brings broader application prospects in the field of service robotss. This model can not only accurately detect human motion keypoints but also achieve posture estimation and behavior recognition in complex environments, providing robots with richer perception and understanding capabilities. This is of great significance for the participation and service provision of service robots in collaborative tasks, such as medical assistance, intelligent transportation, and human-robot cooperation.

## 2 Related work

### 2.1 Based on the top-down human motion pose estimation method

Top-Down human Motion Pose Estimation methods divide human detection and keypoint detection into two stages, effectively integrating global and local information to enhance the accuracy of human motion pose estimation. Among these methods, Simple Baseline is renowned for its simplicity and efficiency, characterized by fast speed, easy implementation, and suitability for real-time applications (Jin et al., 2021; Khirodkar et al., 2021). However, its accuracy may be limited in complex scenarios with significant pose variations. In contrast, Mask-RCNN combines object detection with keypoint detection to improve accuracy and generate semantic pose masks, albeit at the expense of increased computational complexity and slower speed (Ning et al., 2024). On the other hand, Openpose employs a multi-stage convolutional neural network structure for end-to-end human motion pose estimation, particularly excelling in multi-person pose estimation, yet may suffer from inaccurate localization in complex backgrounds (Luo et al., 2021). DEKR enhances accuracy by introducing inter-keypoint correlations, effectively handling occlusions and complex poses, albeit requiring substantial training data and computational resources. CGNet integrates global and local information to improve computational efficiency while maintaining accuracy, but accuracy may decrease in extreme poses and occluded scenarios (Ning et al., 2023). Lastly, PINet achieves a balance between accuracy and speed through staged pose estimation and keypoint refinement strategies, albeit with limited capability in handling complex scenes and small targets (Wang et al., 2023).

These top-down methods, while pursuing higher accuracy, are also striving to improve real-time performance to better adapt to practical applications such as service robots. Current research trends focus on introducing more efficient model structures, optimizing computational processes, and utilizing hardware acceleration to enhance the real-time performance of top-down methods while maintaining accuracy, addressing the needs of service robots and other real-world applications.

### 2.2 Based on the bottom-up human motion pose estimation method

Bottom-Up human motion pose estimation methods adopt a unique strategy by first detecting human body parts in the image and then combining these parts into complete human body poses through effective association algorithms (Cheng et al., 2020). Compared to top-down methods, bottom-up methods are often faster during testing inference, making them particularly suitable for multi-person scenarios. Among them, OpenPose is a classic method that detects human body parts through convolutional neural networks and combines them into complete human body poses using association algorithms, demonstrating strong performance in real-time and multi-person scenario processing. The Associative Embedding (AE) method detects human body parts by generating associative embedding vectors, effectively connecting multiple parts, and enhancing adaptability to complex scenes (Li J. et al., 2020). The Part Affinity Fields (PAF) method utilizes learned human joint affinities to construct affinity fields, aiding in accurately connecting human body parts. HigherHRNet improves the utilization of multiscale information through a hierarchical feature pyramid network, achieving a balance between accuracy and real-time performance. Multiview Pose Machines (MPM), by leveraging multi-view information and synthesizing images from multiple camera angles, provide potential advantages for human motion pose estimation in multi-person collaborative environments. These bottom-up pose estimation methods offer a rich selection of technical choices through different means to address the tasks of motion keypoint detection and quality assessment for service robots, adapting to various scenarios and requirements (Khirodkar et al., 2021; Yao and Wang, 2023).

### 2.3 Research on human motion pose estimation based on YOLO

You Only Look Once (YOLO) is a deep learning model originally designed for real-time object detection, but it has also made significant contributions in the field of human motion pose estimation. In comparison to traditional human motion pose estimation methods, YOLO boasts high real-time performance and lower computational costs, giving it a unique advantage in motion keypoint detection and quality assessment tasks for service robots (Yang et al., 2023).

One of YOLO's key contributions is its end-to-end design, integrating both object detection and human motion pose estimation into a single model. Traditional human motion pose estimation methods often require multiple stages, including human body detection and keypoint localization. YOLO simplifies this process and enhances overall efficiency by directly outputting the target's position and keypoint information through a single forward propagation process. Additionally, YOLO introduces the concept of anchor boxes, using a predefined set of anchor boxes to better adapt to targets of different sizes and proportions (Li et al., 2023). In the context of human motion pose estimation, this means that YOLO can more flexibly handle human bodies of varying sizes and poses, making it more versatile. Another crucial contribution is YOLO's real-time performance. Since service robots typically require quick responses in practical applications, YOLO's high real-time performance makes it an ideal choice for real-time human motion pose estimation. It achieves fast inference speeds through effective model design and optimization without sacrificing accuracy (Liu et al., 2023).

In summary, YOLO's contributions to human motion pose estimation lie primarily in its end-to-end design, the use of anchor boxes, and the achievement of high real-time performance. These features make YOLO a powerful tool, providing an efficient and accurate solution for motion keypoint detection and quality assessment in service robots.

## 3 Method

### 3.1 YOLOv8 network

YOLOv8, the eighth version of “You Only Look Once,” is an advanced object detection model in the YOLO series. Object detection is a fundamental task in computer vision, and YOLOv8 is highly regarded for its excellent balance between accuracy and real-time performance. One of its core features is the adoption of a unified detection framework that allows for simultaneous prediction of multiple objects in an image. In practical applications, YOLOv8 is widely used in autonomous vehicles, surveillance systems, and robotics, among others. Its outstanding real-time performance makes it an ideal choice for scenarios that require fast and accurate object detection.

**Figure 1:**
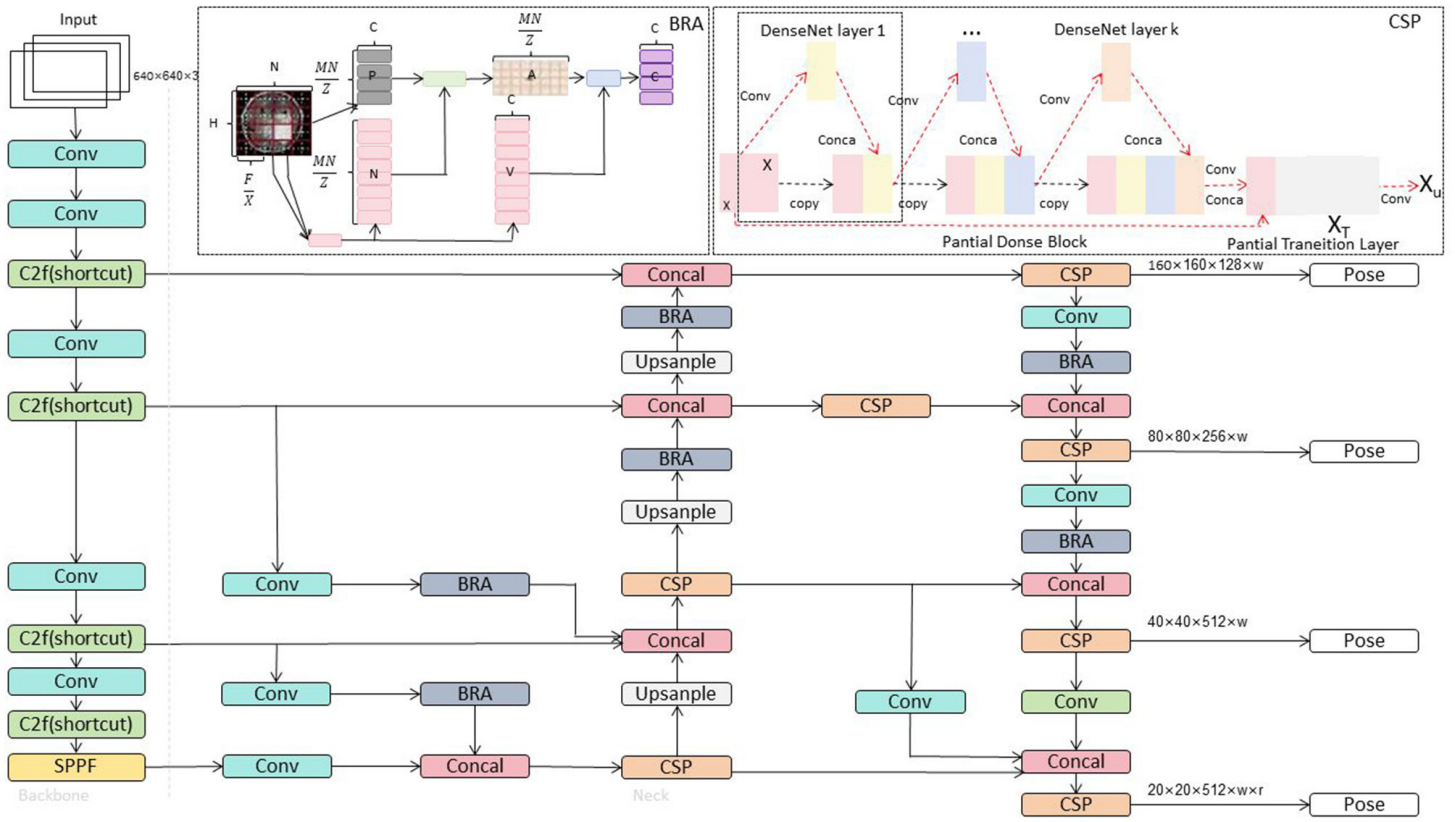
Overall Network architecture diagram of YOLOv8 (Talaat and ZainEldin, 2023).

The overall structure of YOLOv8, as shown in Figure 1, features an optimized backbone architecture using the CSP structure to enhance feature extraction capabilities while maintaining computational efficiency. The model's neck adopts an advanced PAN structure that facilitates the fusion of features from different layers, improving detection performance at various scales. The head of the model uses a decoupled approach, simplifying the prediction process and employing an anchor-free method, contributing to the model's simplicity and efficiency. The loss function in YOLOv8 is a combination of advanced focal loss variants and intersection-over-union (IOU) metrics, fine-tuning the training process to improve model convergence and accuracy. Furthermore, YOLOv8's sample assignment strategy has been improved by using a Task-Aligned Assigner, ensuring that the model's training is more aligned with the specific tasks it needs to perform. This not only makes the model robust but also demonstrates superior generalization capabilities when deployed in real-world scenarios. Training YOLOv8 on large and diverse datasets ensures that the model learns robust features, enabling reliable performance across various settings. Enhancements in data handling, training techniques, and architecture improvements have all contributed to YOLOv8's state-of-the-art performance in the field of object detection.

### 3.2 YOLOv8-ApexNet network

This paper introduces the YOLOv8-ApexNet network as an improved version based on YOLOv8, specifically designed for motion keypoint detection and quality assessment tasks in service robots. YOLOv8 is well-known for its high real-time performance and accurate object detection, and ApexNet builds upon this foundation by introducing two key modules: Generalized Feature Pyramid Network (GFPN) and Bidirectional Routing Attention (BRA).

The GFPN module introduces a pyramid structure, allowing the network to gather multi-scale contextual information at different levels. This improves the network's feature extraction capabilities, enabling it to better adapt to movements of different scales and poses. In motion keypoint detection, this means a more comprehensive understanding of image content, enhancing the accuracy of keypoint localization. The BRA module, through a bidirectional routing mechanism, selectively enhances the network's focus on features at different levels. This mechanism allows the network to concentrate more on critical areas, particularly in complex motion patterns and occlusion scenarios. By guiding attention, BRA increases the network's sensitivity to crucial information, thereby enhancing the detection of motion keypoints. The combined application of these two modules aims to address critical issues in motion keypoint detection and quality assessment tasks for service robots, including improving adaptability to multiple scales and poses and enhancing robustness to complex motion patterns and occlusion.

**Figure 2:**
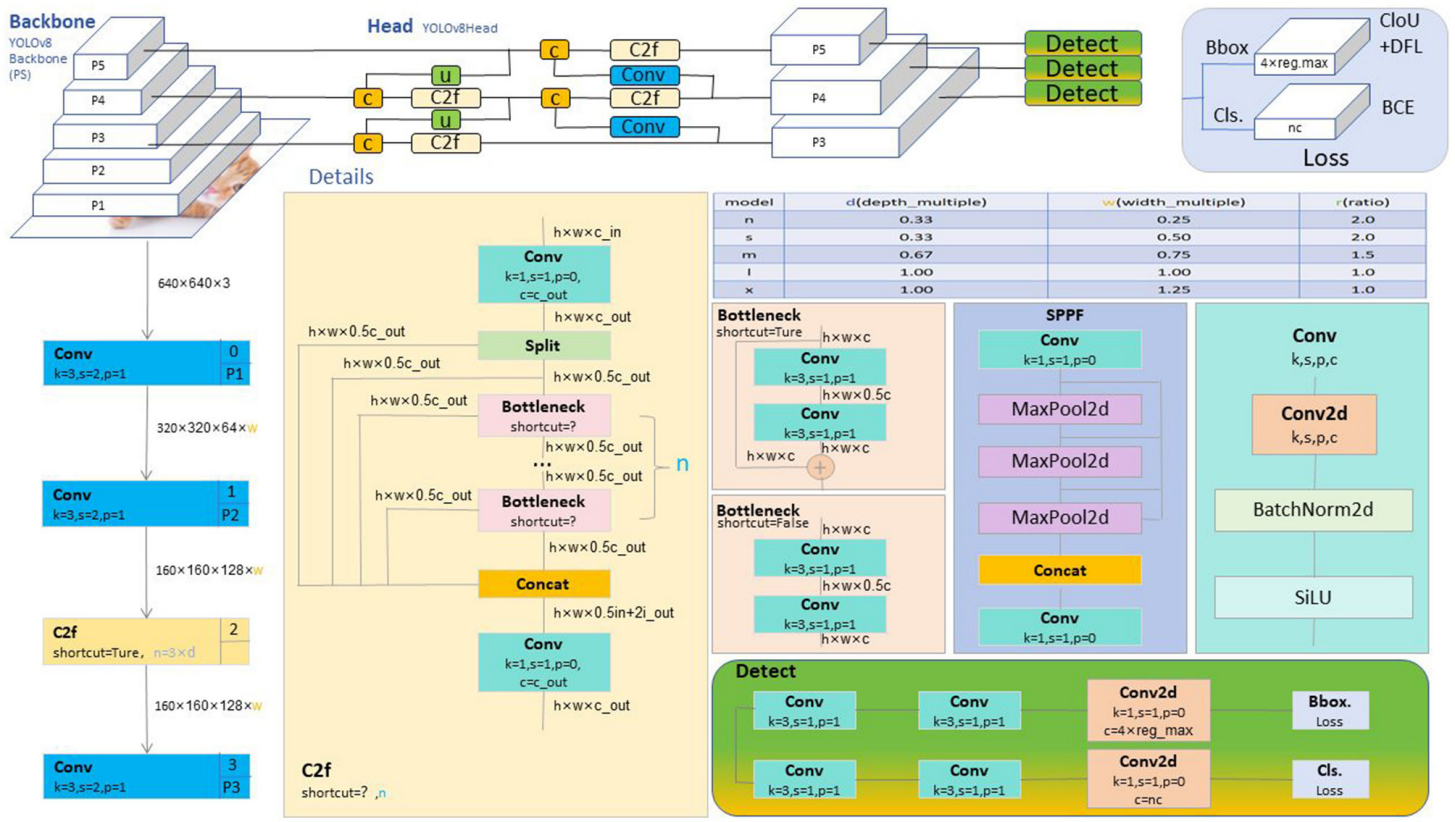
Overall network architecture diagram of YOLOv8-ApexNet.

Through these innovative designs, YOLOv8-ApexNet strives to provide a more accurate and robust solution for the diversity and complexity present in real-world scenarios. The overall network structure of YOLOv8 ApexNet is illustrated in Figure 2.

### 3.3 Generalized Feature Pyramid Network

The Generalized Feature Pyramid Network (GFPN) is a critical technology introduced in the field of deep learning to address the issue of hierarchical feature fusion in Convolutional Neural Networks (CNNs) (Tang et al., 2021). The initially introduced Feature Pyramid Network (FPN) has proven effective in enhancing the performance of deep learning models in object detection tasks, especially when dealing with targets at different scales. The core idea of FPN is to achieve feature hierarchy fusion through both top-down and bottom-up pathways, allowing the network to simultaneously focus on semantic information at different hierarchical levels. This hierarchical fusion helps improve the model's perceptual capabilities for targets at different scales, thereby enhancing the accuracy of object detection. To further strengthen feature propagation and encourage information reuse, improved versions of the Feature Pyramid Network, such as PANet, have been proposed. PANet enhances the representational capability of the feature pyramid by introducing additional pathways and mechanisms, making the network more adaptable to targets with multi-scale structures. Another enhancement is the Bidirectional Feature Pyramid Network (BiFPN), which adds a bottom-up pathway to FPN, enabling bidirectional cross-scale connections. This design effectively leverages multi-scale features, allowing the network to comprehensively perceive the semantic information of targets. The introduction of BiFPN emphasizes further optimization of hierarchical feature fusion, providing a more powerful performance for object detection tasks.

As a key technology for feature fusion, the Generalized Feature Pyramid Network (GFPN) contributes important methodology to enhance the performance of deep learning models in handling multi-scale object detection tasks by extending and improving different versions of the feature pyramid network. In this paper, the introduction of GFPN aims to enhance the perception and processing capabilities of YOLOv8-ApexNet for multi-scale pose information, thereby improving the accuracy of motion keypoints.

In the GFPN formulation, the refined position of each keypoint is updated based on its original position and the weighted sum of displacement vectors from other keypoints.


(1) $$\begin{array}{l} P_{i}^{\prime }=P_{i}+\overset{N}{\underset{j}{\sum }}w_{ij}\cdot \Delta P_{ij} \end{array}$$

where: $P_{i}^{\prime }$ is the refined position of keypoint *i*, *P*_*i*_ is the original position of keypoint *i*, *w*_*ij*_ is the weight between keypoints *i* and *j*, Δ*P*_*ij*_ is the displacement vector from keypoint *i* to keypoint *j*.

In the weight calculation, the weight *w*_*ij*_ is computed based on the exponential scale of the displacement vectors between keypoints *i* and *j*.


(2) $$\begin{array}{l} w_{ij}=\frac{e^{s_{ij}}}{\sum_{k}^{N}e^{s_{ik}}} \end{array}$$

where: *w*_*ij*_ is the weight between keypoints *i* and *j*, *s*_*ij*_ is the scale of the displacement vector from keypoint *i* to keypoint *j*.

The scale *s*_*ij*_ of the displacement vector is predicted through a Multi-Layer Perceptron (MLP) that takes initial scale estimates as input.


(3) $$\begin{array}{l} s_{ij}=\text{MLP}\left(s_{ij}^{\left(0\right)},s_{ij}^{\left(1\right)}\right) \end{array}$$

where: *s*_*ij*_ is the scale of the displacement vector from keypoint *i* to keypoint *j*, $s_{ij}^{\left(0\right)}$ and $s_{ij}^{\left(1\right)}$ are learnable parameters.

The displacement vector Δ*P*_*ij*_ is calculated as the difference between the positions of keypoints *i* and *j*.


(4) $$\begin{array}{l} \Delta P_{ij}=P_{j}-P_{i} \end{array}$$

where: Δ*P*_*ij*_ is the displacement vector from keypoint *i* to keypoint *j*, *P*_*i*_ and *P*_*j*_ are the positions of keypoints *i* and *j*.

The first branch of the scale prediction ($s_{ij}^{\left(0\right)}$) is determined by applying ReLU activation to a linear transformation of the displacement vector.


(5) $$\begin{array}{l} s_{ij}^{\left(0\right)}=\text{ReLU}\left(W_{0}\cdot \Delta P_{ij}\right) \end{array}$$

where: $s_{ij}^{\left(0\right)}$ is the first branch of the scale prediction for keypoints *i* and *j*, *W*_0_ is a learnable weight matrix.

Similarly, the second branch of the scale prediction ($s_{ij}^{\left(1\right)}$) is obtained using another linear transformation and ReLU activation.


(6) $$\begin{array}{l} s_{ij}^{\left(1\right)}=\text{ReLU}\left(W_{1}\cdot \Delta P_{ij}\right) \end{array}$$

where: $s_{ij}^{\left(1\right)}$ is the second branch of the scale prediction for keypoints *i* and *j*, *W*_1_ is another learnable weight matrix.

The final scale prediction through MLP is computed by concatenating the results from the two branches.


(7) $$\begin{array}{l} \text{MLP}\left(x,y\right)=\text{ReLU}\left(W_{2}\cdot \left[x,y\right]+b_{2}\right) \end{array}$$

where: MLP(*x, y*) is a multi-layer perceptron, *x* and *y* are input features, *W*_2_ is a learnable weight matrix, *b*_2_ is a learnable bias vector.

### 3.4 Bidirectional Routing Attention

**Figure 3:**
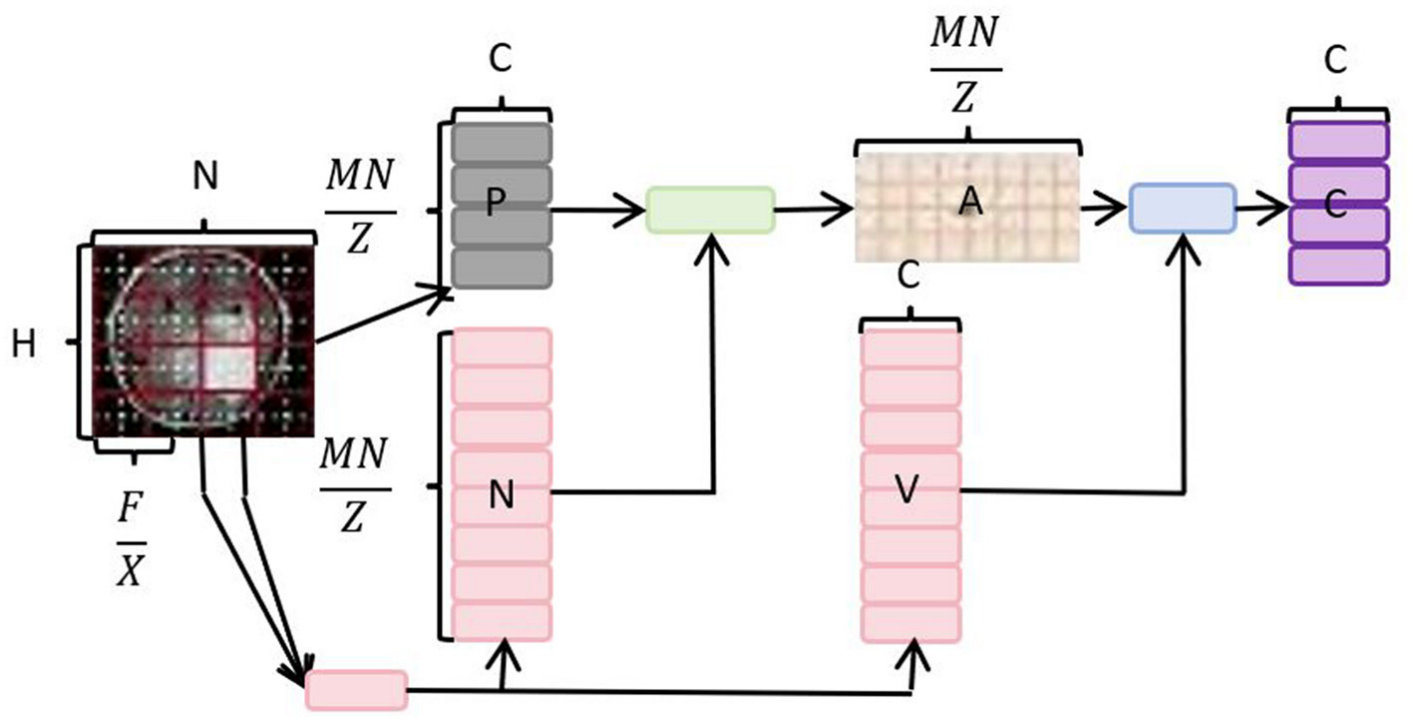
Overall network architecture diagram of BRA.

The core idea of the Neck Multiscale Feature Fusion Network is to merge feature maps extracted from different network layers to enhance the performance of object detection at multiple scales. However, there is a common issue in the feature fusion layer of YOLOv8, namely, the presence of information redundancy from different feature maps (Fang et al., 2022). To overcome this limitation, we introduce a dynamic, query-aware sparse attention mechanism, known as Bidirectional Routing Attention (BRA). As an attention mechanism, BRA provides a small subset of the most relevant keys/values tokens for each query in a content-aware manner. In the feature fusion process of the YOLOv8 model, the introduction of BRA aims to optimize information propagation, reduce information redundancy, and make feature fusion more refined and efficient. This mechanism is dynamic because it adjusts the corresponding keys/values tokens based on the content of the query, allowing the network to flexibly focus on different parts of the features. This is particularly crucial for handling multi-scale object detection tasks, as features at different scales have varying importance for objects of different sizes. In summary, the introduction of Bidirectional Routing Attention (BRA) in the feature fusion layer of YOLOv8 overcomes the issue of information redundancy. Through a dynamic query-aware mechanism, the network intelligently focuses on crucial features, enhancing the performance of multi-scale object detection. The network architecture diagram of BRA is shown in Figure 3.

In the Bidirectional Routing Attention (BRA) mechanism, the query matrix *Q* is obtained by multiplying the input matrix *X* with the learnable query weight matrix *W*_*Q*_.


(8) $$\begin{array}{l} Q=X\cdot W_{Q} \end{array}$$

where: *Q* is the query matrix, *X* is the input matrix, *W*_*Q*_ is the learnable query weight matrix.

The key matrix *K* is derived from the input matrix *X* using the learnable key weight matrix *W*_*K*_.


(9) $$\begin{array}{l} K=X\cdot W_{K} \end{array}$$

where: *K* is the key matrix, *W*_*K*_ is the learnable key weight matrix.

Similarly, the value matrix *V* is calculated by multiplying the input matrix *X* with the learnable value weight matrix *W*_*V*_.


(10) $$\begin{array}{l} V=X\cdot W_{V} \end{array}$$

where: *V* is the value matrix, *W*_*V*_ is the learnable value weight matrix.

The scaled dot-product attention output *S* is computed using the softmax function applied to the normalized dot product of *Q* and *K*^*T*^, divided by the square root of the dimensionality *d*.


(11) $$\begin{array}{l} S=\text{softmax }\left(\frac{Q\cdot K^{T}}{\sqrt{d}}\right)\cdot V \end{array}$$

where: *S* is the scaled dot-product attention output, *d* is the dimensionality of the query and key vectors.

Finally, the output matrix *Y* is obtained by multiplying *S* with the learnable output weight matrix *W*_*O*_.


(12) $$\begin{array}{l} Y=S\cdot W_{O} \end{array}$$

where: *Y* is the final output, *W*_*O*_ is the learnable output weight matrix.

## 4 Experiment

### 4.1 Dataset

The experimental section of this paper is based on two well-known public datasets: Common Objects in Context (COCO) and MPII Human Pose. Additionally, we collected data on athlete pose variations from videos containing various sports activities, encompassing actions such as throwing, running, jumping, and striking.

Firstly, the COCO dataset is a large-scale dataset widely used for object detection and human motion pose estimation, featuring complex images from various daily scenarios (Zhang et al., 2021). The dataset comprises over a million images covering 80 different object categories. For our research, we selected images from the COCO dataset that involve athletes and sports activities to acquire diverse motion pose data.

Secondly, the MPII Human Pose dataset focuses on human motion pose estimation, including images ranging from single individuals to multiple people, along with corresponding annotated keypoints (Zhang et al., 2019). Widely applied in the field of human pose research, this dataset provides detailed pose information for evaluating the model's performance in motion keypoint detection and quality assessment.

By combining the COCO and MPII datasets with self-collected sports activity video data, the experimental section of this paper aims to comprehensively evaluate the performance of service robots motion keypoint detection and quality assessment methods. The goal is to enhance the model's robustness and generalization capabilities across various aspects.

### 4.2 Experimental environment

Hardware Requirements: The server operating system used in this experiment is Ubuntu 20.04.4 LTS. The detailed specifications of the server are as follows: CPU: Intel^®^ Xeon(R) E5–2650 V4@2.20GHz × 48, 128GB RAM, GPU: NVIDIA TITAN V with 12GB of memory. The server configuration meets the computational requirements for the experimental method in this chapter. In the actual experiment, two GPUs were used to enhance training efficiency.

Software Requirements: Python 3.9, PyTorch 1.11.3, CUDA 11.7. PyTorch is a Python-based scientific computing library that primarily implements a series of machine learning algorithms through an executable dynamic computation graph. The use of a dynamic computation graph allows models to be more flexible for adjustment and optimization. PyTorch comes with many optimizers, including SGD, Adam, Adagrad, etc., making it easier for developers to implement optimization algorithms. The specific specifications are shown in Table 1.

**Table 1:** Hardware and software requirements.

**Requirement**	**Specification**
Operating System	Ubuntu 20.04.4 LTS
CPU	IntelⓇ XeonⓇ E52650 V4 @ 2.20GHz × 48
RAM	128GB
GPUs	2 × NVIDIA TITAN V with 12GB memory each
Python version	3.9
PyTorch version	1.11.3
CUDA version	11.7
Features	Dynamic computation graph, multiple optimizers (SGD, Adam, Adagrad)

### 4.3 Baseline

High-resolution network (HRNet) (Seong and Choi, 2021): HRNet is a network architecture based on high-resolution feature maps. In contrast to traditional down-sampling and up-sampling structures, HRNet maintains a flow of high-resolution information, allowing the network to better capture details in poses. The model has achieved significant success in human motion pose estimation tasks, particularly excelling in multi-scale keypoint localization.

HigherHRNet (Cheng et al., 2020): HigherHRNet is an improvement upon HRNet, introducing a hierarchical feature pyramid network. This means the network can simultaneously retain high-resolution information at different levels, effectively enhancing its perception of multi-scale structures. HigherHRNet has demonstrated improved performance in human motion pose estimation, especially when dealing with scenes involving complex multi-scale variations.

YoloV5Pose (Hou et al., 2020): YoloV5Pose is a human motion pose estimation model based on YoloV5, leveraging YoloV5's object detection capabilities and extending them to human motion pose estimation tasks. The model adopts a single-stage detection approach, integrating object detection and keypoint localization for more efficient end-to-end training and inference. YoloV5Pose strikes a balance between speed and accuracy, making it suitable for real-time scenarios.

YoloV8pose (Liu et al., 2023): YOLOv8pose is an upgrade from YOLOv5Pose. This model utilizes deep learning techniques to detect key keypoints of the human body in a single image, enabling real-time prediction of human body poses. By leveraging multi-scale features and advanced network architecture, YOLOv8pose can accurately capture complex human poses and achieve higher performance and robustness across various scenarios.

OpenPose (Chen et al., 2020): OpenPose is a classic multi-person human motion pose estimation framework that simultaneously detects multiple keypoints using convolutional neural networks. The model performs feature extraction at multiple levels, effectively capturing spatial relationships in human poses. OpenPose has set benchmarks in the field of open human motion pose estimation and is widely applied to various real-time human analysis tasks.

Hourglass (Xu and Takano, 2021): Hourglass is a recursive network structure that accomplishes multi-scale modeling of poses through multi-level bottom-up and top-down processing. Inspired by the hourglass structure of the human body, the model efficiently handles complex relationships in human poses. Hourglass has demonstrated outstanding performance in image semantic segmentation and human motion pose estimation tasks.

LightOpenPose (Zhao et al., 2022): LightOpenPose is a lightweight optimized version of OpenPose, aiming to maintain accuracy while reducing the model's computational complexity. Through a series of lightweight designs and network optimizations, LightOpenPose delivers acceptable performance even in resource-constrained environments. This makes it practically feasible for embedded systems and mobile applications.

### 4.4 Implementation details

#### 4.4.1 Data processing

All images in the dataset have been labeled and then converted into the YOLO format for storage. This process ensures that key points or motion targets in each image are accurately identified. Labeling can be done using manual annotation tools or through automated computer vision algorithms. Subsequently, the labeled image data is converted into the YOLO format, which includes information such as the category of each object, the center coordinates of the bounding box, and its width and height. This format conversion ensures that the data matches the input format required for model training.

This experimental dataset contains 9,210 images, and the dataset is divided to provide three different subsets for training, validation, and testing. The division follows a 70-15-15 ratio, with 70% of the data used for training, 15% for validating the performance of the model, and the remaining 15% for testing the model's generalization ability. Reasonable data division allows for a better assessment of the model's training status and accurate evaluation of its performance on unseen data.

Data normalization is carried out to ensure that the model better handles images of different scales and brightness during training. The image data is normalized, scaling pixel values to a range of 0 to 1. At the same time, the bounding box coordinates in the YOLO format are also normalized by dividing the center coordinates, width, and height by the width and height of the image, bringing their values between 0 and 1. This helps the model better understand the relative position of the bounding boxes.

#### 4.4.2 Network parameter setting

The initial step in preprocessing the input images is to adjust the length of the longer side to a predetermined target size, ensuring a consistent aspect ratio among different images. To achieve this, we adopted a strategy where the image is resized to the target dimensions, and padding is applied on the shorter side to form a square image. This approach ensures that all input images have a uniform size of 640 × 640 pixels, providing a consistent data shape for subsequent model input.

To enhance the robustness of the network, we introduced various data augmentation techniques. First, we applied horizontal flipping to expand the dataset and increase the model's robustness to mirrored poses. Second, we employed multi-scale adjustment techniques, randomly varying the size of the images (within a range of 20%) to further increase the model's adaptability to poses at different scales. Techniques such as random translation (within a range of 2%) and random rotation (within a range of 35%) were also incorporated to simulate pose variations that might occur in real-world scenarios, thereby improving the model's generalizability (Sattler et al., 2019). In the final 10 stages of training, we adopted a strategy of disabling these data augmentation techniques. This approach ensures that the model focuses on learning more refined features as it nears convergence, achieving higher accuracy and robustness. This strategy enables us to develop a human motion pose estimationons.

Specific training parameters can be found in Table 2. They were determined through careful tuning and experimentation to ensure that the model adequately learns key points and motion features in the images. The choice of these training parameters was meticulously designed to balance the complexity of the model and its learning effectiveness, aiming for optimal training results.

**Table 2:** Model training parameters.

**Parameter**	**Settings**
Optimizer	SGD
Learning rate	0.01
Batch size	32
Epoch	200
Input size	640 × 640

#### 4.4.3 Evaluation metrics

In this paper, we primarily employ classic evaluation metrics widely used in object detection tasks to comprehensively assess the performance of our proposed robot motion keypoint detection method. Specifically, we focus on the following key evaluation metrics:

Average Precision at 50% Intersection over Union (AP50): the Average Precision at 50% Intersection over Union (AP50) is a crucial metric in object detection evaluation. It measures the accuracy of the model by considering the precision and recall at a 50% IoU threshold. The formula is given by:


(13) $$\begin{array}{c} {\text{AP}}^{50}=\frac{1}{\left|C\right|}\overset{\left|C\right|}{\underset{i=1}{\sum }}\text{Precision}\left(R_{i},P_{i},0.5\right)\times \text{Recall}\left(R_{i},G_{i},0.5\right) \end{array}$$

where: |*C*|: the number of object classes. *R*_*i*_: the set of detected bounding boxes for class *i*. *P*_*i*_: the set of ground truth bounding boxes for class *i*. Precision(*R*_*i*_, *P*_*i*_, 0.5): Precision at 50% IoU for class *i*. Recall(*R*_*i*_, *G*_*i*_, 0.5): Recall at 50% IoU for class *i*.

Average Precision at 75% Intersection over Union (AP^75^): The Average Precision at 75% Intersection over Union extends the evaluation to a stricter 75% IoU threshold. It provides a more stringent assessment of model performance. The formula is expressed as:


(14) $$\begin{array}{c} {\text{AP}}^{75}=\frac{1}{\left|C\right|}\overset{\left|C\right|}{\underset{i=1}{\sum }}\text{Precision}\left(R_{i},P_{i},0.75\right)\times \text{Recall}\left(R_{i},G_{i},0.75\right) \end{array}$$

where the variables have the same meaning as in AP^50^.

Average Precision (Medium)—AP^*M*^: The Average Precision (Medium) or AP^*M*^ focuses on the performance of the model concerning objects of medium size. The formula is defined as:


(15) $$\begin{array}{c} {\text{AP}}^{M}=\frac{1}{\left|C\right|}\overset{\left|C\right|}{\underset{i=1}{\sum }}\text{AP}\left(R_{i},P_{i},\text{Medium}\right) \end{array}$$

where AP(*R*_*i*_, *P*_*i*_, Medium) denotes the Average Precision with medium-sized objects for class *i*.

Average Precision (Large)—AP^*L*^: Similarly, the Average Precision (Large) or AP^*L*^ assesses the model's accuracy with respect to large-sized objects. The formula is given by:


(16) $$\begin{array}{c} {\text{AP}}^{L}=\frac{1}{\left|C\right|}\overset{\left|C\right|}{\underset{i=1}{\sum }}\text{AP}\left(R_{i},P_{i},\text{Large}\right) \end{array}$$

where AP(*R*_*i*_, *P*_*i*_, Large) represents the Average Precision with large-sized objects for class *i*.

We utilize the mean deviation as a measure for assessing the pivotal angle and incorporate a margin of tolerance τ, recognizing that minor discrepancies are permissible in the practical identification of pivotal points. The JAM is determined under the tolerance threshold:


$$JAM=1-\frac{\sum_{i=1}^{n}\max\left(0,|y_{i}-Y_{i}|-\tau \right)}{\sum_{i=1}^{n}y_{i}}$$

Where, *y*_*i*_ represents the calculated joint angle, *Y*_*i*_ denotes the reference value, τ stands for the tolerance limit, *i* signifies the *i*-th predicted joint angle, and *n* denotes the total number of joint angles (sample size).

### 4.5 Results

As shown in Table 3, we conducted comparative experiments to evaluate the performance of different methods on the COCO and MPII datasets. The table presents the performance of each method across various evaluation metrics (AP^50^, AP^75^, AP^*M*^, AP^*L*^). Firstly, our method achieved the highest performance across all evaluation metrics on the COCO dataset. Specifically, compared to other methods, our approach outperformed them by 3.1%, 8.2%, 3.4%, and 3.8% in AP^50^, AP^75^, AP^*M*^, and AP^*L*^, respectively. This indicates that our method exhibits higher accuracy and robustness in object detection and human motion pose estimation tasks. On the MPII dataset, our method similarly demonstrated the best performance. Compared to the best results of other methods, our approach improved by 3.2%, 8.4%, 3.7%, and 3.8% in AP^50^, AP^75^, AP^*M*^, and AP^*L*^, respectively. This further confirms the outstanding performance of our method in the motion keypoint detection and quality assessment tasks for robots.

**Table 3:** Performance comparison of methods on COCO and MPII datasets.

		**COCO datasets**				**MPII datasets**			
**Methods**	**Backbone**	**AP^50^**	**AP^75^**	**AP^*M*^**	**AP^*L*^**	**AP^50^**	**AP^75^**	**AP^*M*^**	**AP^*L*^**
HRNet (Seong and Choi, 2021)	HRNet-W32	86.83	55.43	61.13	75.53	84.65	53.25	58.95	73.35
HigherHRNet (Cheng et al., 2020)	HRNet-W48	87.43	55.93	61.23	75.93	85.25	53.85	59.05	73.75
YoloV5pose (Hou et al., 2020)	Darknet-csp-d53-s	87.03	61.23	61.53	76.33	85.85	59.05	59.35	74.15
YoloV8pose (Liu et al., 2023)	Darknet-csp-d53-s	88.04	61.58	61.73	75.23	86.87	60.15	58.48	75.37
Openpose (Chen et al., 2020)	————	86.13	56.73	60.23	74.73	83.95	54.55	58.05	72.55
Hourglass (Xu and Takano, 2021)	Hourglass	85.73	55.43	58.93	72.13	83.55	53.25	56.75	69.85
Lightopenpose (Zhao et al., 2022)	————	81.33	54.13	58.63	70.53	79.15	51.95	56.45	68.35
Ours	Darknet-53	89.93	63.63	64.63	78.73	87.75	61.45	62.45	76.55

Our method excelled on both datasets, showing significant performance improvements compared to other methods. This demonstrates that our proposed approach has higher adaptability and generalization capabilities in real-world applications, providing an efficient and accurate solution for motion keypoint detection and quality assessment in robots.

**Figure 4:**
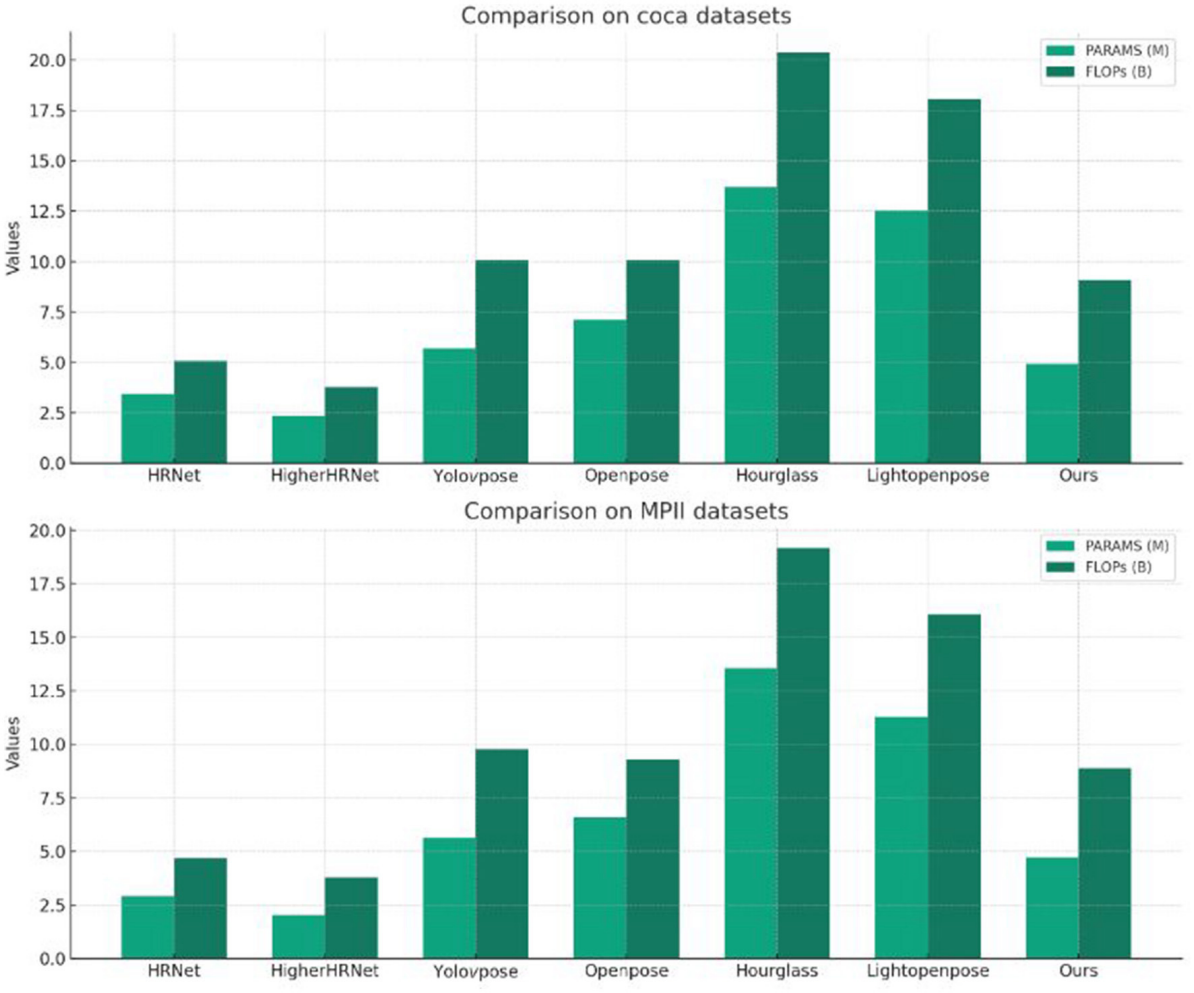
Comparison of model performance on different datasets.

Table 4 presents a comparison of model parameters (PARAMS) and floating-point operations (FLOPs) among different models on the COCO and MPII datasets. On the COCO dataset, our model has PARAMS of 4.93M and FLOPs of 9.08B. Compared to other methods, our model achieves significant reductions in both parameter count and computational complexity, by 28.0 and 11.1%, respectively. This indicates that our model maintains satisfactory performance while keeping a lower computational burden. On the MPII dataset, our model also excels in PARAMS and FLOPs, with reductions of 26.5 and 6.5%, respectively. Although our model may not be optimal in terms of parameter count and computational complexity, this performance still balances higher accuracy and robustness with satisfactory computational efficiency. The result of the table visualization is shown in Figure 4.

**Table 4:** Comparison of model parameters (PARAMS) and floating point operations (FLOPs) on COCO and MPII datasets.

**Model**	**COCO Dataset**		**MPII dataset**	
	**PARAMS**	**FLOPs**	**PARAMS**	**FLOPs**
HRNet (Li Y. et al., 2020)	3.43M	5.08B	2.91M	4.68B
HigherHRNet (Cheng et al., 2020)	2.35M	3.78B	2.03M	3.78B
YoloV5pose (Hou et al., 2020)	5.70M	10.08B	5.65M	9.78B
Openpose (Chen et al., 2020)	7.11M	10.08B	6.61M	9.28B
Hourglass (Xu and Takano, 2021)	13.71M	20.38B	13.56M	19.18B
Lightopenpose (Zhao et al., 2022)	12.53M	18.08B	11.28M	16.08B
Ours	4.93M	9.08B	4.73M	8.88B

The reason our model performs better in terms of computational complexity (FLOPs) is due to the optimization of network structure and the introduction of efficient modules, which reduces the number of floating-point operations required when processing images. Specifically, we adopted a lightweight network design and efficient feature extraction modules to decrease the computational load for each image processing. Additionally, we conducted fine-tuning of the network structure to minimize unnecessary computational burdens while maintaining good performance. Overall, our approach provides a competitive solution for motion keypoint detection and quality assessment tasks in robotics. Future work can further optimize the model structure to achieve a better balance between performance and computational efficiency.

### 4.6 Ablation experiment

As shown in Table 5, we conducted ablation experiments to systematically evaluate the impact of introducing different components (BRA and GFPN) on the performance of the model in the task of robotic motion keypoint detection and quality assessment. The four different methods in the table correspond to the following scenarios: (1) the baseline model, which is the basic model without BRA and GFPN, and its performance is evaluated on the COCO and Post datasets; (2) the model with BRA, to study the singular impact of BRA on performance; (3) the model with GFPN, to study the singular impact of GFPN on performance; (4) the model with both BRA and GFPN, to comprehensively assess the joint impact of these two components on performance.

**Table 5:** Ablation experiment results on COCO and MPII datasets.

			**COCO datasets**				**MPII datasets**			
**Method**	**BRA**	**GFPN**	**AP^50^**	**AP^50 − 95^**	**AP^*M*^**	**AP^*L*^**	**AP^50^**	**AP^50 − 95^**	**AP^*M*^**	**AP^*L*^**
(1)			88.23	62.33	63.53	77.13	86.05	60.15	61.35	74.95
(2)	✓		89.23	62.93	64.43	78.43	87.05	60.75	62.25	76.25
(3)		✓	89.73	63.53	64.83	77.23	87.55	61.35	62.65	75.05
(4)	✓	✓	89.93	64.73	64.63	78.73	87.75	63.15	62.45	76.55

We utilized multiple performance evaluation metrics (AP^50^, AP^50 − 95^, AP^*M*^, and AP^*L*^) to compare the model performance under various conditions. The experimental results clearly demonstrate that the introduction of both BRA and GFPN significantly enhances the model's performance. Particularly noteworthy is that when introducing both BRA and GFPN simultaneously, the model achieves the best performance across all evaluation metrics. This finding further confirms the crucial role of BRA and GFPN in robotic motion keypoint detection and quality assessment tasks. These experimental results provide solid support for the effectiveness of our proposed method in practical applications and offer valuable insights for future optimizations of model structures to achieve superior performance.

### 4.7 Presentation of results

In the evaluation of motion scene fitting, our method demonstrates outstanding superiority, as shown in Table 6. Taking the tennis scene as an example, our model achieves a remarkable center joint fitting accuracy (JAM^*c*^) of 90.5%, showcasing higher joint accuracy compared to other motion scenes. This result is further validated across other joints, including the left joint (JAM^*s*^) and right joint (JAM^*k*^), with accuracies of 88.2 and 87.0%, respectively. Simultaneously, our model exhibits excellent performance in the original missed detection rate (MR), ensuring its reliability in real-world scenarios. It is noteworthy that our method excels not only in the tennis scene but also in various motion scenes such as football, skiing, gymnastics, and running. This encompasses a comprehensive improvement in joint fitting accuracy and original missed detection rate in football scenes, demonstrating the robustness and high accuracy of our method across diverse motion scenes.

**Table 6:** JAM is the fitted accuracy, and MR is the original missed detection rate.

**Type**	**JAM^*c*^**	**JAM^*s*^**	**JAM^*k*^**	**MR^*c*^**	**MR^*s*^**	**MR^*k*^**
Tennis	90.5	88.2	87.0	10.4	11.6	14.6
Football	86.1	84.4	80.2	16.7	15.5	17.3
Skiing	88.4	88.3	89.5	16.3	14.1	13.8
Gymnastics	95.5	94.0	93.6	11.3	10.2	13.4
Running	92.3	88.7	88.9	12.9	13.9	18.6

**Figure 5:**
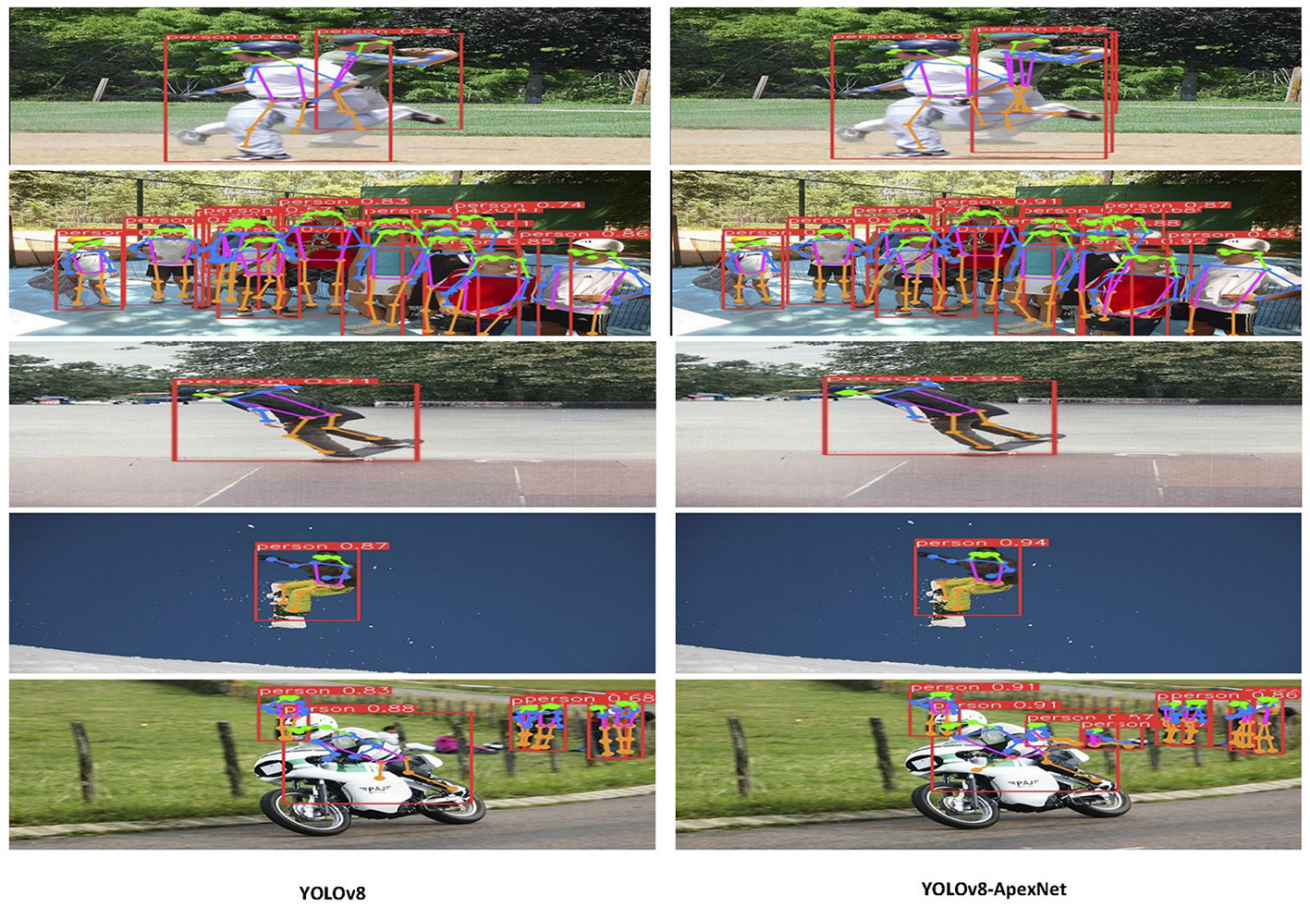
Performance analysis of YOLOv8-ApexNet detection and YOLOv8 detection results comparison.

As shown in Figure 5, we conducted a detailed comparison of the performance between YOLOv8-ApexNet and YOLOv8 in real-world motion scenarios. The experimental results indicate that our model excels in various aspects, demonstrating significant advantages over YOLOv8. Firstly, in terms of occlusion, YOLOv8-ApexNet exhibits stronger tolerance compared to YOLOv8. Our model, utilizing the Bidirectional Routing Attention (BRA) technology, successfully captures inter-keypoint correlations in dynamic scenes, thereby enhancing its ability to recognize occluded objects. In contrast, YOLOv8 may experience significant interference when dealing with occluded scenes, leading to a decline in target detection performance. Secondly, in handling small targets, YOLOv8-ApexNet maintains high detection accuracy even for small target sizes. With the incorporation of the Generalized Feature Pyramid Network (GFPN) technology, our model effectively extracts and integrates feature information across different scales, enabling better adaptation to various target sizes and shapes. Conversely, YOLOv8 may experience a performance decline in small target detection. Lastly, in terms of confidence estimation, YOLOv8-ApexNet demonstrates more reliable and accurate target confidence assessment in the experiments. Through optimized algorithms and network structures, our model achieves a significant improvement in predicting target confidence, making it more stable and precise compared to YOLOv8. Through comparative experiments in real-world motion scenarios, our YOLOv8-ApexNet model excels in occlusion handling, small target detection, and target confidence estimation, providing a more reliable and accurate solution for practical applications in target detection.

## 5 Conclusion

This study proposes an innovative model through an in-depth exploration of robot motion key point detection and quality assessment tasks. In experimental evaluations, our model demonstrates outstanding performance on the COCO and MPII datasets, achieving significant improvements over other methods in key point localization accuracy and model robustness. Through ablation experiments, we validate the positive impact of introducing BRA and GFPN on model performance, showcasing excellent performance in different motion scenarios. Visualizations of tables and figures further support the superiority of our approach in real-world scenarios, providing an effective solution for robot motion key point detection.

However, despite the significant achievements of our model, there are still some shortcomings. Firstly, in certain complex scenarios, the model may lack robustness in handling key point occlusion or abnormal poses. Secondly, the adaptability to specific motion scenarios needs further improvement to meet a wider range of practical application requirements.

Looking ahead, we are committed to further optimizing the model's robustness and adaptability. Firstly, by introducing more training data from complex scenarios and designing more sophisticated loss functions, we aim to enhance the model's ability to handle key point occlusion and abnormal poses. Secondly, we plan to expand the model's applicability to different motion scenarios, achieving better generalization performance through more flexible structural designs. Additionally, we will explore the application of the model in real robotic systems to validate its feasibility in practical engineering tasks. In summary, the robot motion key point detection model proposed in this study demonstrates significant advantages in experiments, providing a valuable reference for future in-depth research and practical applications. By addressing challenges in real-world problems, our work is poised to contribute more practical and innovative solutions to the field of robotics perception and decision-making.

## Data availability statement

The raw data supporting the conclusions of this article will be made available by the authors, without undue reservation.

## Author contributions

XT: Data curation, Investigation, Project administration, Visualization, Writing – original draft. SZ: Conceptualization, Methodology, Project administration, Visualization, Writing – review & editing.
